# Correlations among neck circumference and anthropometric indicators to estimate body adiposity in people living with HIV

**DOI:** 10.1590/0037-8682-0649-2020

**Published:** 2021-03-08

**Authors:** Natália Alves Oliveira, Nathalia Sernizon Guimarães, Samantha Luiza Mazon e Silva, Anny Carolina Messias, Gabriela Fonseca Lopes, Israel Borges do Nascimento-Júnior, Sidney Augusto Vieira-Filho, Rachel Basques Caligiorne, Sônia Maria de Figueiredo

**Affiliations:** 1 Universidade Federal de Ouro Preto, Programa de Pós-Graduação em Saúde e Nutrição, Ouro Preto, MG, Brasil.; 2 Medical College of Wisconsin, School of Medicine, Wisconsin, Milwaukee, United States of America.; 3 Universidade Federal de Ouro Preto, Departamento de Farmácia, Ouro Preto, MG, Brasil.; 4 Hospital Santa Casa de Belo Horizonte, Instituto de Ensino e Pesquisa, Belo Horizonte, MG, Brasil.

**Keywords:** Anthropometry, Fat Body, HIV, Neck

## Abstract

**INTRODUCTION:**

Neck circumference (NC) and anthropometric data of people living with HIV (PLWH) are correlated.

**METHODS:**

Socioeconomic, NC, body mass index (BMI), tricipital skinfold thickness (TSF), mid-arm circumference (MAC), mid-arm muscle circumference (MAMC), waist-hip ratio (WHR), waist-to-height ratio (WHtR), waist circumference (WC), and hip circumference (HC) data of 72 PLWH were correlated.

**RESULTS:**

Higher adiposity was observed in NC (40.3% [n=29]) and WC (31.9% [n=23]). Correlations between NC/BMI, NC/WC, NC/HC, NC/MAC, NC/MAMC, and NC/WHtR were significant. Increased NC (40.3%[n=29]) and WC (31.9 [n=23]) were associated with higher cardiometabolic risk.

**CONCLUSIONS:**

NC correlations are adequate for estimating cardiometabolic risk.

Human immunodeficiency virus/acquired immune deficiency syndrome (HIV/AIDS) is challenging to treat. In 2019, 1,700,000 new people were diagnosed with HIV^1^. People living with HIV (PLWH) are administered antiretroviral therapy (ART) involving a combination of two or more medications to control the viral load. This clinical therapy has reduced the mortality rates and enhanced the life expectancy of PLWH^1^. However, in recent years, ART has been associated with metabolic and nutritional disturbances such as glucose homeostasis changes, dyslipidemia enhancement, and body composition changes and increased risks of comorbidities such as cardiovascular diseases^2^.

Interest in associating the neck circumference (NC) with anthropometric data to estimate body fat has increased in recent years^3^. Representative samples of adult and older people (aged 18-70 years) of both sexes not infected by HIV have shown that NC values correlate with anthropometric indicators of non-visceral, abdominal, and/or visceral fat and total body fat mass^4^ ^,^ ^5^. However, there is insufficient relevant data to state that this association remains in the presence of HIV. To contribute to this evaluation process, the correlation of NC with anthropometric indicators in PLWH and its correlation with body fat was studied.

A cross-sectional study involving 72 PLWH aged 19-73 years was conducted at a primary health care facility in the city of Ouro Preto, MG, Brazil. OpenEpi software was used to calculate the sample size based on the following parameters: (a) number of PLWH aged above 18 years in Ouro Preto (n=102), (b) lipodystrophy prevalence ≥84%, (c) 5% variation, and (d) confidence interval ≥95%. The minimum sample size was 69 patients. People with confirmed HIV aged over 18 years who were receiving continued ART and those who agreed to participate and provided a written informed consent form were included. The participants who fulfilled the inclusion criteria were invited to participate in the study from May 2018 to October 2019. Pregnant women and people with impaired mental conditions, which impeded participation, were excluded. The research was approved by the Ethics Committee of the Federal University de Ouro Preto (CAAE-14135913.7.0000.5150), and all interviews were individualized and conducted in private practice.

Data on triglyceride, total cholesterol, high-density lipoprotein (HDL), low-density lipoprotein (LDL), and fasting glycemia levels; CD4+ T lymphocyte count; viral load; and ART strategy were obtained from medical records^6^.

On survey completion, the interviewers performed a physical examination to obtain the anthropometric indicator data. An inextensible anthropometric tape (1.5 m length, 0.1 mm resolution) was used to measure body circumferences. Body height was measured using a mechanical stadiometer, body weight was measured using a platform weighing scale, and the skin folds were measured using an adipometer^7^. Waist circumference (WC) was determined at the largest abdominal perimeter between the last rib and iliac crest. WC >80 cm for females and WC >94 cm for males, indicative of central obesity, were considered high^8^. Hip circumference (HC) assessment was performed at the largest perimeter of the hip. The mid-arm circumference (MAC) was obtained at the midpoint between the acromion and the olecranon, and the values were evaluated as suggested by Frisancho^9^. The mid-arm muscle circumference (MAMC) was evaluated using the following equation: MAMC (%) = MAC (cm)-[π x (TSF (mm) ÷ 10)], where the π value is 3.14 and TSF stands for the triceps skin fold^8^ ^,^ ^10^. The type of fat distribution was evaluated by the waist-to-hip ratio (WHR) using the equation: WHR = WC (cm) ÷ hip (cm). Central obesity parameters characterized by WHR >0.85 in females and WHR >1.0 in males were considered indicative of a high amount of fat. The waist-to-height ratio (WHtR) was obtained using the equation: WHtR = WC (cm) ÷ height (cm). WHtR >0.50 was considered representative of a high cardiometabolic risk state^7^ ^,^ ^8^. Body mass index (BMI) was estimated as follows: body weight (kg) ÷ [height (m)]^2^. In the nutritional status evaluation, individuals aged <60 years and individuals aged >60 years were considered^7^. NC was determined at the midpoint of the neck with an individual standing in the orthostatic position and a horizontal plane. The NC measurement in males was measured just below the Adam’s apple on the cricoid cartilage. A cut-off point of ≥34 for females and ≥ 37 for males was considered increased^11^. The energy intake and food groups were evaluated using a 24-h reminder^12^.

All data were exported to EpiData version 3.1 and analyzed using IBM SPSS version 18.0. Categorical variables are represented by descriptive statistics, either considering an absolute (n) or relative (%) value, and the continuous variables are represented as the mean and standard deviation (SD). The categorized variables were compared and evaluated using Pearson’s chi-squared test.

The normality and distribution of the continuous variables were verified using the Shapiro-Wilk test. The Mann-Whitney U test and Student’s t-test were used to analyze the mean difference for continuous variables non-parametric and parametric data, respectively. Pearson’s (parametric data) correlation test was used to analyze the correlations of NC with the anthropometric indicators of body fat. A significance level of 0.05 was used.

The participants included in this study were 72 PLWH with an average age of 42±12 years. Males constituted the majority of the sample (males: 65.3%, n=47; females: 34.7%, n=25).

The mean CD4+ T lymphocyte counts were within the normal range, and most patients (77.8%, n=56) had undetectable viral loads (<50 copies/mL). The levels of total cholesterol (p<0.020) and LDL (p<0.005) were significantly higher in females than in males. However, no significant differences in HDL levels were found between sexes. Dyslipidemia was found in 61.8% (n=34) participants (Table 1).

The PLWH reported that they ate all their meals and that they consumed fruit, juices, raw salads, whole-meal cereals, and bread. However, the energy intake was significantly different between males and females (p<0.001; Table 1).

Based on BMI, 45.8% (n=33) PLWH were classified as eutrophic and 40.3% (n=29) were classified as overweight. The prevalence of tricipital skinfold thickness (TST; 62.5%), MAC (43.1%), and MAMC (81.9%) were observed, indicating that these PLWH were underweight (Table 1). Compared to females, males showed higher values related to average height, body weight, and NC. However, females showed higher TST values (Table 1). Based on WHtR, most PLWH (52.8%) were classified as high risk of body fat. However, based on WHR, 77.8% (n=56) PLWH were classified as having a low risk of cardiovascular disease. For both WHR and WHtR, a significant difference between sexes was found (Table 1). The NC (40.3%, n=29) and WC (31.9%, n=23) were positively correlated with excess body fat . The value of WC indicates a higher prevalence of central obesity in females. A similar prevalence was observed for WC and WHtR, indicating that central obesity was significantly higher in females than in males (Table 1). Comparing the WHtR and NC of males and females, there were no significant differences related to the prevalence of excess body fat (Table 1).

**TABLE 1:** Clinical, biochemical characteristics, energy intake, and anthropometric indicators related to the sex of people living with human immunodeficiency virus in Ouro Preto, Minas Gerais, Brazil (2019).

Variables	Total (N=72)		Females (n=25)		Males (n=47)		***p*-value**
	N	%	n	%	n	%	
**Clinical characteristics**							
CD4^+^ T lymphocyte count, mean (95% CI)	544.7 (478.13-611.21)		516.0 (419.86-612.14)		559.91(469.53-650.30)		0.732*
Undetectable viral load	56	77.8	20	80.0	36	76.6	0.741
Time of HIV infection in years, mean (95% CI)	6.25 (5.08-7.42)		8.20 (6.32-10.08)		5.21 (3.77-6.65)		0.224*
Time of ART in years, mean (95% CI)	5.31( 4.28-6.33)		6.40 (5.07-7.73)		4.72(3.32-6.12)		0.210*
**Biochemical characteristics**							
Triglycerides (mean±SD)	120.61±53.33		119.81±48.98		121.00±55.97		0.939
Total cholesterol (mean±SD)	181.85±39.84		199.61±32.76		173.21±40.50		0.020
LDL (mean±SD)	108.70±33.59		126.51±34.75		100.03±29.77		0.005
HDL (mean±SD)	51.88±16.21		47.85±12.52		53.83±17.56		0.202
Mean fasting glycemia (CI 95%)	111.69 (78.61-144.77)		96.24 (84.07-108.41)		119.63 (69.28-169.97)		0.634*
**Energy intake**							
Energy intake. kcal (mean±SD)	1825.65±678.72		1439.80±456.31		2030.90±691.68		0.000
**Anthropometric indicators**							
Meters high (mean±SD)	1.66±0.11		1.55±0.08		1.72±0.07		0.000^¥^
Body mass kg (mean±SD)	68.69±16.25		61.91±16.45		72.30±15.10		0.009^¥^
BMI kg/m² (mean±SD)	24.6±5.06		25.65±6.60		24.04±3.99		0.202^¥^
**BMI classification**							
*Underweight*	10.0	13.9	4.0	16	6.0	12.8	0.473**
*Eutrophy*	33.0	45.8	9.0	36	22.0	51.1	
*Overweight/Obesity*	29.0	40.3	12.0	48	17.0	36.2	
TSF mm (mean±SD)	13.6±7.46		17.98±7.57		11.27±6.34		0.000^¥^
**TSF classification**							
*Underweight*	45.0	62.5	16.0	64	29.0	61.7	0.375**
*Eutrophy*	5.0	6.9	3.0	12	26.0	4.3	
*Overweight/Obesity*	22.0	30.6	6.0	24	16.0	34	
MAC cm (mean±SD)	28.75±6.35		28.82±9.49		28.72±3.91		0.950^¥^
**MAC classification**							
*Underweight*	31	43.1	12	48	19	40.4	0.334**
*Eutrophy*	28	38.9	7	28	12	44.7	
*Overweight/Obesity*	13	18.1	6	24	7	14.9	
**MAMC classification**							
*Underweight*	59	81.9	16	64	43	91.5	0.004**
*Eutrophy*	13	18.1	9	36	4	8.5	
NC cm (mean±SD)	35.58±3.86		32.73±3.19		37.1±3.31		0.001^¥^
**NC Classification**							
*Regular*	43.0	59.7	17.0	68	62.0	55.3	0.296**
*Increased*	29.0	40.3	8.0	32	12.0	44.7	
WC cm (mean±SD)	83.99±13.10		83.45±17.40		84.28±10.33		0.801^¥^
High-risk WC	32.0	31.9	14.0	56	9.0	19.1	0.001**
WHR (mean±SD)	0.87±0.09		0.87±0.11		0.87±0.08		0.808^¥^
High-risk WHR	16.0	22.2	14.0	56	2.0	4.3	0.000**
WHtR (mean±SD)	0.51±0.08		0.54±0.09		0.49±0.05		0.013^¥^
High-risk WHtR	38.0	52.8	17.0	68	21.0	44.7	0.059**

Note: **CI:** confidence interval, **ART:** antiretroviral therapy, **SD:** standard deviation, **LDL:** low-density lipoprotein, **HDL:** high-density lipoprotein, **BMI:** body mass index, **TSF:** tricipital skinfold, **MAC:** mid-arm circumference, **MAMC:** mid-arm muscle circumference. Student’s t-test-parametric variables; * Mann-Whitney U test-non-parametric variables; **Pearson’s Chi-square test. The p-value refers to the difference between males and females.

Correlations of the NC with other anthropometric indicators of body fat were examined (Figure 1). Except for TST and WHR, there was a significant positive correlation between NC and other indicators. A high correlation of the NC with body weight and a median association between the NC and height was observed (r=0.700; p<0.001). However, a low-intensity correlation was observed between the NC with BMI, WC, and HC (Figure 1).

Individually, the values of anthropometric indicators can lead to different conclusions related to the nutritional status of PLWH. However, comparing the NC data with the data of other anthropometric indicators, the following positive correlations were established: NC/BMI, NC/WC, NC/HC, NC/MAC, and NC/WHR (Figure 1). BMI data indicated a high proportion of eutrophic (45.8%) and overweight (40.3%) PLWH.

**FIGURE 1:**
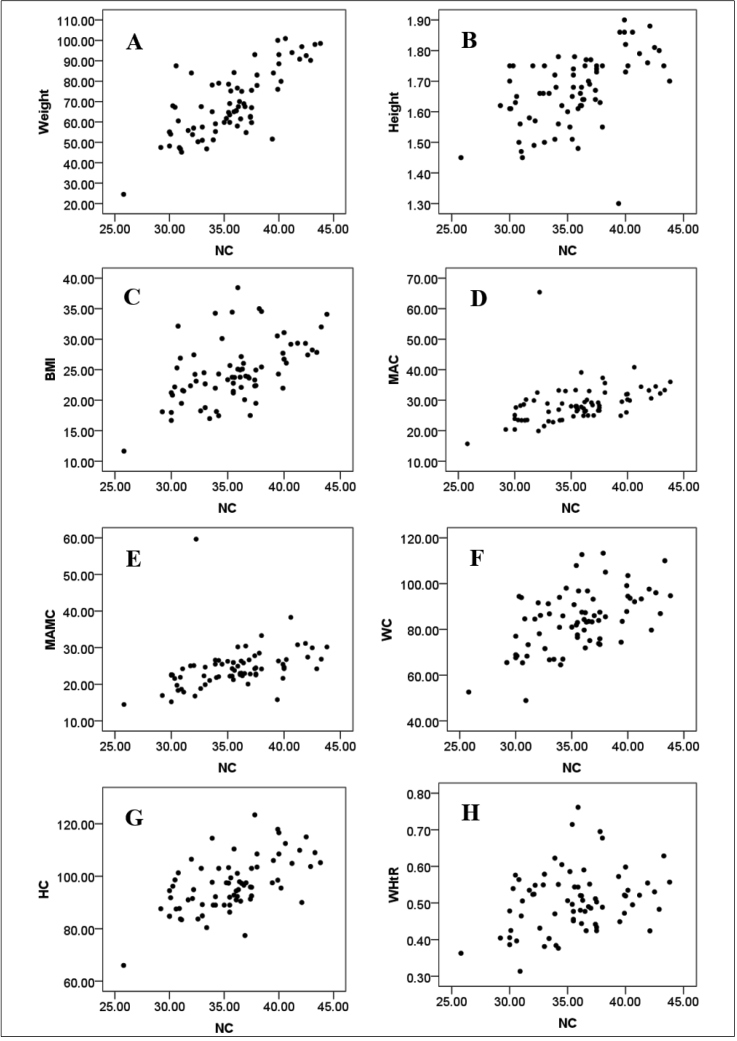
Scatter plot between neck circumference (NC) and the anthropometric parameters of nutritional status evaluations of people living with human immunodeficiency virus in Ouro Preto, Minas Gerais, Brazil (2019). **(A)** Weight *vs.* NC (r=0.700; p<0.001); **(B)** Height *vs*. NC (r=0.553, p<0.001); **(C)** Body mass index (BMI) *vs.* NC (r=0.526, p <0.001) **(D)** mid arm circumference (MAC) *vs.* NC (r=0.555, p<0.001); **(E)** mid-arm muscle circumference (MAMC) *vs.* NC (r=0.564, p<0.001); **(F)** waist circumference (WC) *vs.* NC (r=0.501, p<0.001) (**G**) hip circumference (HC) *vs.* NC (r=0.577, p<0.001), and waist-to-hip ratio (WHtR) *vs.* NC (r=0.258, p <0.05). r: Pearson correlation coefficient.

Considering the percentage of TST (62.5%), MAC (43.1%), and MAMC (81.9%), a high frequency of undernutrition was found. These findings are in agreement with the results reported by Pires et al.^6^. The malnutrition found in our study may be related to HIV lipodystrophy, masking this clinical case with excess body fat. HIV-associated lipodystrophy, characterized by endocrine-metabolic changes and body fat distribution, was observed in the studied population. The diagnostic significance of being underweight among the evaluated PLWH could be overestimated due to the influence of lipoatrophy, characterized by subcutaneous fat loss in peripheral regions^13^. In line with this result, we found a high frequency of dyslipidemia (61.8%) in our study participants. ART can control the physiology of PLWH, inducing morphological changes^13^ in the body.

Correlations between NC and BMI, WC, and HC indicated a proportionality between the changes in body adiposity and fat accumulation in the subcutaneous region of the neck in the PLWH. These correlations are in accordance with prior studies involving non-HIV-infected people^14^ ^,^ ^15^ and contribute to validating the NC as an adequate parameter for predicting body fat. Data on the use of NC in adults and older PLWH is scarce, demonstrating the need for studies to include this indicator as an additional tool for the nutritional assessment of PLWH^15^. 

Regarding food consumption in PLWH, we observed that males had a higher energy intake than females. However, females had higher total cholesterol, LDL, and triglyceride levels than men, suggesting that this females possibly consume more fatty foods. This result highlights the need for nutritional status monitoring to avoid malnutrition and potential cardiometabolic risks, regardless of sex^4^ ^,^ ^5^.

Anthropometry is an essential tool for diagnosing nutritional status and monitoring the health of PLWH. We suggest that NC measurements can be used to evaluate the nutritional status of PLWH and estimate excessive body fat in this population.

## References

[1] World Health Organization (2020). Global Health Observatory data repository. Number of new HIV infections.

[2] Ministério da Saúde. Departamento de Vigilância, Prevenção e Controle das IST, do HIV/Aids e das Hepatites Virais (2020). Brazil. Painel de Indicadores Epidemiológicos.

[3] Oliveira NA, Figueiredo SM, Guimarães NS (2019). A medida da circunferência do pescoço pode ser usada como indicador de adiposidade corporal? revisão sistemática. Revista Brasileira de Obesidade, Nutrição e Emagrecimento.

[4] Alfadhli EM, Sandokji AA, Zahid BN, Makkawi MA, Alshenaifi RF, Thani TS (2017). Neck circumference as a marker of obesity and a predictor of cardiometabolic risk among Saudi subjects. Saudi Med J.

[5] Albassam RS, Lei KY, Alnaami AM, Al-Daghri NM (2019). Correlations of neck circumference with body composition and cardiometabolic risk factors in Arab women. Eat Weight Disord.

[6] Pires DS, Ferraz SF, Monteiro ML, Reis VAGA, Pontes DB, Andrade MI (2017). Nutritional profile and methods for assessing the nutritional status of HIV-infected patients. Braspen J.

[7] World Health Organization (WHO) (1995). Physical status: the use and interpretation of anthropometry.

[8] Lohman TG, Roche AF, Martorell R (1988). Anthropometric standardization reference manual.

[9] Frisancho AR (1990). Anthropometric Standards for the assessment of growth and nutricional status.

[10] World Health Organization (WHO) (2008). Waist circumference and waist-hip ratio: report of a WHO expert consultation, Geneva, 8-11 December 2008.

[11] Ben-Noun L, Sohar E, Laor A (2001). Neck circumference as a simple screening measure for identifying over weight and obese patients. Obes Res.

[12] Subar AB, Thompson FE, Potischman N, Forsyth BH, Buday R, Richards D (2007). Formative research of a quick list for an automated selfadministered 24-hour dietary recall. J Am Diet Assoc.

[13] Dragović G, Danilović D, Dimić A, Jevtović D (2014). Lipodystrophy induced by combination antiretroviral therapy in HIV/AIDS patients: a Belgrade cohort study. Vojnosanit Pregl.

[14] Fitch KV, Stanley TL, Looby SE, Rope AM, Grinspoon SK (2011). Relationship Between Neck Circumference and Cardiometabolic Parameters in HIV-Infected and non-HIV-Infected Adults. Diabetes Care.

[15] Luo Y, Ma X, Shen Y, Xu Y, Xiong Q, Zhang X (2017). Neck circumference as an effective measure for identifying cardio-metabolic syndrome: a comparison with waist circumference. Endocrine.

